# Genetic Variants at 10p11 Confer Risk of Tetralogy of Fallot in Chinese of Nanjing

**DOI:** 10.1371/journal.pone.0089636

**Published:** 2014-03-03

**Authors:** Jing Xu, Yuan Lin, Linjie Si, Guangfu Jin, Juncheng Dai, Cheng Wang, Jiaping Chen, Min Da, Yuanli Hu, Chenlong Yi, Zhibin Hu, Hongbing Shen, Xuming Mo, Yijiang Chen, Xiaowei Wang

**Affiliations:** 1 Department of Thoracic and Cardiovascular Surgery, The First Affiliated Hospital of Nanjing Medical University, Nanjing, China; 2 State Key Laboratory of Reproductive Medicine, School of Public Health, Nanjing Medical University, Nanjing, China; 3 Department of Epidemiology and Biostatistics and Key Laboratory of Modern Toxicology of Ministry of Education, School of Public Health, Nanjing Medical University, Nanjing, China; 4 Department of Cardiothoracic Surgery, Nanjing Children's Hospital, Nanjing Medical University, Nanjing, China; MOE Key Laboratory of Environment and Health, School of Public Health, Tongji Medical College, Huazhong University of Science and Technology, China

## Abstract

A recent genome-wide association study (GWAS) has identified a new subset of susceptibility loci of Tetralogy of Fallot (TOF), one form of cyanotic congenital heart disease (CHD), on chromosomes 10p11, 10p14, 12q24, 13q31, 15q13 and 16q12 in Europeans. In the current study, we conducted a case-control study in a Chinese population including 1,010 CHD cases [atrial septal defect (ASD), ventricular septal defect (VSD) and TOF] and 1,962 controls to evaluate the associations of these loci with risk of CHD. We found that rs2228638 in *NRP1* on 10p11 was significantly increased the risk of TOF (OR = 1.52, 95% CI = 1.13–2.04, *P* = 0.006), but not in other subgroups including ASD and VSD. In addition, no significant associations were observed between the other loci and the risk of ASD, VSD or TOF. Our results suggested that the genetic variants on 10p11 may serve as candidate markers for TOF susceptibility in Chinese population.

## Introduction

Congenital heart disease (CHD) is the most common birth defect and represents the leading cause of infant morbidity, affecting 4 to 10 per 1000 live births [1]. Despite tremendous progress in diagnosis, prevention, and management of CHD, the exact etiology of CHD is still largely unknown. Over the past decade, molecular genetic studies have demonstrated that only about 20% of CHD are due to either chromosomal conditions or multisystem malformation syndromes. The remaining 80% of ‘sporadic’ cases have been considered to be multifactorial inheritance that involves a multitude of susceptibility genes with low-penetrance mutations (common variants) or intermediate-penetrance mutations (rare variants) superposed onto unfavorable environmental factors [2], [3].

Recently, genome-wide association studies (GWAS) have provided a systematic way to search for genetic variants and successfully identified multiple low-penetrance susceptibility loci for various complex diseases, which have greatly improved our understanding of the genetic basis of human diseases [4], [5]. In 2013, a two-stage GWAS of Tetralogy of Fallot (TOF), one form of cyanotic CHD, was performed by Cordell *et al.* in Europeans population, in which 835 TOF patients and 5,159 controls were scanned using the genome-wide single nucleotides polymorphism (SNP) chips, and the SNPs with *P*≤1×10^−5^ were further evaluated in additional 798 TOF patients and 2,931 controls. As a result, genetic variants at 10p11, 10p14, 12q24, 13q31, 15q13 and 16q12 were identified to be significantly associated with risk of TOF [6]. These findings may present new insight into the etiology of TOF. However, these new identified loci of TOF have not yet been validated in other populations to date, especially in non-European populations. In addition, the broad phenotypic spectrum of CHD suggests a complex underlying genetic network with a large number of different modifier genes. Since the GWAS by Cordell *et al*. only focused on the susceptibility of TOF, it is currently unclear whether these loci have effects on other phenotypes of CHD.

In the current study, with an effort to evaluate the relationships of genetic variants at 10p11, 10p14, 12q24, 13q31, 15q13 and 16q12 with the risk of CHD in non-European populations, we conducted an independent case-control study with 1,010 CHD cases and 1,962 controls in a Chinese population and genotyped seven marker SNPs (rs1857231, rs2228638, rs734186, rs4771856, rs12593223, rs6499100 and rs233716) to test the associations with the risk of CHD. In addition, we also looked up the association results of these SNPs in our existing GWAS dataset reported previously [7].

## Materials and Methods

### Ethics statement

This study was approved by the institutional review board of Nanjing Medical University. The design and performance of current study involving human subjects were clearly described in a research protocol. All participants and/or their parents were voluntary and would complete the informed consent in written before taking part in this research.

### Study population

In this study, 1,010 cases with ASD, VSD or TOF and 1,962 controls were recruited from the First Affiliated Hospital of Nanjing Medical University and the Affiliated Nanjing Children's Hospital of Nanjing Medical University (Nanjing, China) between March 2009 and May 2013. Non-syndromic CHD cases were diagnosed on the basis of echocardiography and were further confirmed by cardiaccatheterization and/or surgery. Cases having clinical features of developmental syndromes, multiple major developmental anomalies or known chromosomal abnormalities were excluded. Cases were also excluded if they had a positive family history of CHD in a first-degree relative (parent, sibling or child), maternal diabetes mellitus, phenylketonuria, maternal exposure to teratogens (for example, from pesticides and organic solvents) or maternal exposure to therapeutic drugs during the intrauterine period. Controls were outpatients without CHD from the same geographic areas. They were recruited from the hospitals above during the same time period. Controls with congenital anomalies or cardiac disease were excluded. The controls were frequency-matched to the cases based on age and gender. All subjects were genetically unrelated individuals of Han Chinese ancestry. For each participant, approximately 2 ml of whole blood was obtained to extract genomic DNA for genotyping analysis.

### SNP selection and genotyping

Based on the findings from the GWAS of TOF in Europeans, 18 genetic variants were significantly associated with TOF risk (Table S1). Considering the differences of minor allele frequencies (MAF) and linkage disequilibrium (LD) structures between Chinese and Europeans, the SNPs selection followed three criteria: (a) reported marker SNPs in TOF GWAS; (b) minor allele frequency (MAF)≥0.05 in Chinese Han Beijing (CHB) based on the HapMap database; (c) only one SNP with the lowest *P* value was selected when multiple SNPs showed a strong LD (r^2^≥0.8) (Table S2). As a result, seven SNPs (rs1857231, rs2228638, rs734186, rs4771856, rs12593223, rs6499100 and rs233716) were selected for this study (Table 1).

**Table 1:** Association results of 7 SNPs with CHD risk.

Chr.	SNPs	Study	Cases b	Controls b	MAF c		OR_add_ (95% CI) d	*P*_add_ d
					Cases	Controls		
10p14	rs1857231	All cases	21/284/700	61/554/1325	0.16	0.17	0.92(0.79–1.06)	0.24
	A/G a	ASD	12/109/244	61/554/1325	0.18	0.17	1.06(0.86–1.30)	0.60
		VSD	4/121/306	61/554/1325	0.15	0.17	0.83(0.68–1.02)	0.08
		TOF	5/54/150	61/554/1325	0.15	0.17	0.86(0.65–1.13)	0.28
10p11.22	rs2228638	All cases	15/197/790	24/316/1622	0.11	0.09	1.24(1.04–1.47)	**0.01**
	G/A a	ASD	4/66/295	24/316/1622	0.10	0.09	1.10(0.85–1.42)	0.47
		VSD	8/81/342	24/316/1622	0.11	0.09	1.23(0.97–1.55)	0.08
		TOF	3/50/153	24/316/1622	0.14	0.09	1.52(1.13–2.04)	**0.006**
10p11.22	rs734186	All cases	46/316/629	71/641/1247	0.21	0.20	1.04(0.91–1.19)	0.59
	T/C a	ASD	16/111/231	71/641/1247	0.20	0.20	1.00(0.82–1.22)	0.99
		VSD	20/147/256	71/641/1247	0.22	0.20	1.14(0.95–1.37)	0.16
		TOF	10/58/142	71/641/1247	0.19	0.20	0.91(0.70–1.18)	0.49
12q24.13	rs233716	All cases	137/450/395	260/878/820	0.37	0.36	1.05(0.94–1.17)	0.39
	G/A a	ASD	41/169/145	260/878/820	0.35	0.36	0.99(0.83–1.16)	0.86
		VSD	59/188/171	260/878/820	0.37	0.36	1.04(0.89–1.21)	0.63
		TOF	10/58/142	71/641/1247	0.40	0.36	1.19(0.97–1.46)	0.09
13q31.3	rs4771856	All cases	105/458/441	229/873/846	0.33	0.34	0.96(0.86–1.08)	0.49
	C/A^a^	ASD	34/172/158	229/873/846	0.33	0.34	0.95(0.80–1.12)	0.53
		VSD	48/186/196	229/873/846	0.33	0.34	0.94(0.80–1.10)	0.44
		TOF	23/100/87	229/873/846	0.35	0.34	1.03(0.83–1.27)	0.81
15q13.3	rs12593223	All cases	69/395/537	157/754/1018	0.27	0.28	0.95(0.84–1.07)	0.39
	G/A^a^	ASD	29/131/203	157/754/1018	0.26	0.28	0.92(0.77–1.10)	0.37
		VSD	31/169/228	157/754/1018	0.27	0.28	0.97(0.82–1.14)	0.68
		TOF	9/95/106	157/754/1018	0.27	0.28	0.96(0.77–1.21)	0.74
16q12.2	rs6499100	All cases	51/306/648	98/646/1218	0.20	0.21	0.93(0.82–1.07)	0.31
	C/T a	ASD	19/119/229	98/646/1218	0.21	0.21	1.00(0.82–1.20)	0.97
		VSD	21/126/284	98/646/1218	0.19	0.21	0.89(0.74–1.07)	0.21
		TOF	11/61/135	98/646/1218	0.20	0.21	0.92(0.72–1.18)	0.51

a Major/minor alleles;

b Minor homozygote/Heterozygote/Major homozygote;

c Minor allele frequency;

d OR (95% CI) and *P* value derived from logistic regression analysis in additive model.

Genomic DNA was extracted from a leukocyte pellet by proteinase K digestion and followed by phenol-chloroform extraction and ethanol precipitation. The seven SNPs (rs1857231, rs2228638, rs734186, rs4771856, rs12593223, rs6499100 and rs233716) were genotyped by the TaqMan allelic discrimination Assay on an ABI 7900 system (Applied Biosystems, Foster City, CA). The primers and probes were shown in Table S3. A series of methods were used to control the quality of genotyping: (i) case and control samples were mixed on each plate; (ii) genotyping was performed without knowing the case or control status; (iii) two water controls were used in each plate as blank control.

### Statistical analyses

Distribution differences of demographic characteristics and genotypes between the cases and controls were analyzed using *χ^2^* test or student t test. Agreement with Hardy-Weinberg equilibrium was tested using a goodness-of-fit *χ^2^* test among the control subjects. The associations of genotypes/alleles with CHD risk were estimated by computing odds ratios (ORs) and 95% confidence intervals (CIs) in an additive model from logistic regression analyses with an adjustment for age, and sex. All statistical analyses were performed with Statistical Analysis System software (v.9.1.3; SAS Institute, Cary, NC).

## Results

The characteristics of CHD cases and non-CHD controls for this study were shown in Table S5. There were no significant differences for the distributions of age and gender between the cases and controls (*P* = 0.38 and 0.94, respectively). Among 1,010 CHD cases, there were 367 (36.3%) atrial septal defects (ASD) cases, 432 (42.8%) ventricular septal defects (VSD) cases and 211 (20.9%) TOF cases.

The genotype distributions of the seven selected SNPs (rs1857231, rs2228638, rs734186, rs4771856, rs12593223, rs6499100 and rs233716) between the cases and controls were shown in Table 1. The observed genotype frequencies of seven variants were in agreement with the Hardy-Weinberg equilibrium among the controls (*P*>0.05 for all SNPs). The results showed that the variant genotypes of rs2228638 at 10p11 was significantly associated with an increased risk of CHD [additive model: odds ratio (OR) = 1.24, 95% confidence interval (CI) = 1.04–1.47, *P* = 0.01], which was more evident for TOF [additive model: OR = 1.52, 95% CI = 1.13–2.04, *P* = 0.006]. The association of rs2228638 with TOF risk was still significant after bonferroni correction (*P* = 0.042). However, no significant associations were observed between rs2228638 and other subgroups of CHD [additive model: OR = 1.10, 95% CI = 0.85–1.42, *P* = 0.47 for ASD; OR = 1.23, 95% CI = 0.97–1.55, *P* = 0.08 for VSD]. Moreover, we did not find significant associations for the other six SNPs with the risk of overall CHD or subtypes (*P*>0.05) (Table 1).

In order to provide further information on the association of these seven SNPs with CHD risk, we also checked the existing GWAS [7] with 945 CHD cases (including 334 ASD, 534 VSD and 77 ASD/VSD) and 1,246 controls in Chinese population. As shown in Table S4, we did not observe significant associations between these seven SNPs with the risk of overall CHD or ASD and VSD (*P*>0.05).

## Discussion

In this study, we examined the genetic variants at 10p11, 10p14, 12q24, 13q31, 15q13 and 16q12, which were associated with TOF risk in Europeans, in a case-control study with 1,010 CHD cases and 1,962 controls in a Chinese population. We confirmed the association of the locus 10p11with TOF risk but not for other loci. We further find that all of these seven loci were not significantly associated with the risk of ASD and VSD, which was also supported by a previous GWAS dataset in Chinese population. These findings suggest the potential heterogeneity of TOF susceptibility between ethnicities as well as heterogeneous etiology for CHD subtypes.

TOF is the most common form of cyanotic congenital heart defect, accounting for approximately 10% of all CHD, with an incidence of approximately 3 of every 10,000 live births [8]. The precise cause of TOF is unknown. Most cases seem sporadic, although the risk of recurrence in siblings is about 3% if there are no other affected first-degree relatives [9]. Improved understanding of possible causes may provide insight into the pathobiological basis of the TOF and facilitate to predict disease risk [10]. In recent years, several genes associated with monogenic forms of non-syndromic TOF have been reported, including those encoding human homolog of the Drosophilia jagged protein JAG1[11]–[13], GATA family zinc finger transcription factors GATA4 [14], [15] or GATA6 [16], [17], T-box transcription factor TBX1 [18], [19] and Notch family translocation-associated type 1 transmembrane receptor NOTCH1 [20], [21]. In contrast, GWAS may be more useful in clarifying the TOF related genes in low penetrance.

The polymorphism rs2228638 is a nonsynonymous coding SNP that results in the substitution of Isoleucine for Valine at position 733 of the neuropilin-1 protein, which is encoded by the gene *NRP1*. In the GWAS of TOF by Cordell *et al*., the authors reported that individuals carrying variant A allele of rs2228638 had a 1.28-fold risk of TOF than those with the wild G allele. In this study, we firstly replicated this association in a non-European population with an increased TOF risk of 1.52-fold for A allele carriers, indicating that this SNP may also be a susceptibility marker for TOF in Chinese population.

The neuropilin NRP1 is identified as a multifunctional transmembrane glycoprotein receptor with a small cytoplasmic domain and multiple extracellular domains capable of mediating a variety of protein/protein interactions which also plays a vital role in cardiovascular development [22], [23]. NRP1 is expressed in multiple cell types that contribute to development of the cardiovascular system including cardiac neural crest cells and endothelial cells [24], [25]. Targeted disruption of NRP1 gene has demonstrated an essential role of this molecule in cardiovascular development. NRP1-null mice die between E12 and E13.5 with a spectrum of cardiovascular defects characterized by lack of development of the dorsal aorta, transposition of the aortic arch and insufficient septation of the truncus arteriosus [26]. The NRP1 mutant mice also exhibit multiple cardiac defects including persistent truncus arteriosus, misplacement of the coronary arteries and ventricular septal defects due to deficiencies of both NRP1-Sema signaling and NRP1-VEGF signaling [27]. These findings suggest that the dysfunction of NRP1 is implicated in the failure septation of the cardiac outflow tract and ventricular septal defect which are the main structural abnormalities in TOF development. However, the mechanism through which the genetic variants at 10p11 affect the development of TOF is still largely unknown, though the SNP rs2228638 is a nonsynonymous SNP with potential function. Therefore, future investigation is needed to illustrate whether the variant rs2228638 could alter the structure and function of neuropilin-1 which might influence NRP1-Sema signaling and NRP1-VEGF signaling in the subtle temporal and spatial regulation processes that occur during heart development.

CHD usually refers to abnormalities in the heart's structure and can be classified into three broad categories: cyanotic heart disease, left-sided obstruction defects, and the more common septation defects, but the proportion of each category varies greatly in different geographical regions [28]. The first GWAS study have identified multiple low-penetrance susceptibility loci and these loci may provide novel insights into cardiac development, as the phenotypic analysis merely confined to TOF, whether these loci correlated with other phenotypes of CHD remains unclear. In the present study, we tried to extend the associations from TOF to the more common septation defects including atrial septal defects and ventricular septal defects in Chinese population. However, this hypothesis was not supported both in our study and pervious GWAS of CHD in Chinese populations [7]. In spite of ethnic difference, these findings may further demonstrate the heterogeneity of etiology among subtypes of CHD.

In the present study, several limitations need to be addressed. First, we recruited CHD cases and controls from two hospitals in Nanjing area, which might not well represent the whole population and might result in potential selection bias. Second, CHD comprised of broad phenotypic spectrum, we only replicated the potential positive SNPs in TOF and extended to septation defects, other phenotypes of CHD were not included in this study. Third, the biological mechanism of genetic variants at 10p11were not further investigated in this study. It is important for future studies with larger samples and functional characterizations to validate our findings.

In summary, our study represents an independent replication study for the reported GWAS of TOF in European populations. We clearly showed that the locus rs2228638 at 10p11 was also associated with TOF risk in Chinese populations and this SNP may be useful as genetic marker in risk prediction of TOF in Chinese.

## Supporting Information

Table S1 **Information of SNPs associated with the risk of Tetralogy of Fallot in Europeans reported by Cordell et al.**
(DOC)

Table S2 **SNPs in strong linkage disequilibrium (r2≥0.8) with selected ones.**
(DOC)

Table S3 **The primers and probes for detection the 7 selective SNPs.**
(DOC)

Table S4 **Associations of 7 SNPs with CHD in previous GWAS in Chinese population.**
(DOC)

Table S5 **Characteristics of CHD cases and non-CHD controls.**
(DOC)
