# Supplementary material for: Genetic Variants at 10p11 Confer Risk of Tetralogy of Fallot in Chinese of Nanjing
Source: PLoS One. 2014 Mar 3;9(3):e89636. doi: 10.1371/journal.pone.0089636 (PMC3940663; doi:10.1371/journal.pone.0089636)
Supplement: Table S1 — Information of SNPs associated with the risk of Tetralogy of Fallot in Europeans reported by Cordell et al. (DOC) [file pone.0089636.s001.doc]

**Table S1:** Information of SNPs associated with the risk of Tetralogy of Fallot in Europeans reported by Cordell et al.

| **Chr.** | **SNP** | **Position** | **Major/Minor Allele** | **MAF ^a^** |
| --- | --- | --- | --- | --- |
| 10p14 | rs2388896 | 8954224 | A/G | 0.19 |
| 10p14 | rs1857231 | 8961614 | A/G | 0.19 |
| 10p11.22 | rs2228638 | 33475282 | A/G | 0.09 |
| 10p11.22 | rs734186 | 33484829 | C/T | 0.10 |
| 12q24.12 | rs3184504 | 111884608 | C/T | 0.01 |
| 12q24.12 | rs653178 | 112007756 | A/G | 0 |
| 12q24.12 | rs11065987 | 112072424 | A/G | 0 |
| 12q24.13 | rs17696736 | 112486818 | A/G | 0 |
| 12q24.13 | rs11066188 | 112610714 | A/G | 0 |
| 12q24.13 | rs11066320 | 112906415 | A/G | 0 |
| 12q24.13 | rs233722 | 113031474 | C/T | 0.38 |
| 12q24.13 | rs233716 | 113039943 | A/G | 0.37 |
| 13q31.3 | rs7982677 | 92988323 | A/C | 0.28 |
| 13q31.3 | rs4771856 | 92994509 | A/C | 0.32 |
| 15q13.3 | rs12593223 | 33092962 | A/G | 0.22 |
| 16q12.2 | rs1420258 | 52821637 | A/G | 0.16 |
| 16q12.2 | rs6499100 | 52831462 | C/T | 0.16 |
| 16q12.2 | rs1579237 | 52833422 | C/T | 0.16 |

^a^ Minor allele frequency in Chinese Han Beijing (CHB) based on the HapMap database.
