# Supplementary material for: Genetic Variants at 10p11 Confer Risk of Tetralogy of Fallot in Chinese of Nanjing
Source: PLoS One. 2014 Mar 3;9(3):e89636. doi: 10.1371/journal.pone.0089636 (PMC3940663; doi:10.1371/journal.pone.0089636)
Supplement: Table S2 — SNPs in strong linkage disequilibrium (r2≥0.8) with selected ones. (DOC) [file pone.0089636.s002.doc]

**Table S2:** SNPs in strong linkage disequilibrium (r2 ≥ 0.8) with selected ones

| **Chr.** | **Position** | **SNP** | **Position** | **SNP** | **r^2 a^** |
| --- | --- | --- | --- | --- | --- |
| 10p14 | 8954224 | rs2388896 | 8961614 | rs1857231 | 1.00 |
| 12q24.13 | 113039943 | rs233716 | 113031474 | rs233722 | 0.92 |
| 13q31.3 | 92994509 | rs4771856 | 92988323 | rs7982677 | 0.84 |
| 16q12.2 | 52831462 | rs6499100 | 52821637 | rs1420258 | 1.00 |
| 16q12.2 | 52821637 | rs1420258 | 52833422 | rs1579237 | 1.00 |
| 16q12.2 | 52831462 | rs6499100 | 52833422 | rs1579237 | 1.00 |

^a^ Linkage disequilibrium analysis based on our previous GWAS in Chinese populaiton
