# Supplementary material for: Genetic Variants at 10p11 Confer Risk of Tetralogy of Fallot in Chinese of Nanjing
Source: PLoS One. 2014 Mar 3;9(3):e89636. doi: 10.1371/journal.pone.0089636 (PMC3940663; doi:10.1371/journal.pone.0089636)
Supplement: Table S3 — The primers and probes for detection the 7 selective SNPs. (DOC) [file pone.0089636.s003.doc]

**Table S3:** The primers and probes for detection the 7 selective SNPs

| **SNP** | **F-primer** | **R-primer** | **FAM-probe** | **HEX-probe** |
| --- | --- | --- | --- | --- |
| rs1857231 | CTCACATTTAATACTTTCCGATCTTGTTC | GGTGAATATTATGTTGTGCTTACTATAGAAAGG | FAM-TGCTTCCATTGCCT-MGB | HEX-TGCTTCCGTTGCCT-MGB |
| rs2228638 | GCATGACCTTCTGGTATCACATG | GCTTCTGGTAGCGCAGTTTGA | FAM-TCCCACGTCGGCAC-MGB | HEX-TCCCACATCGGCAC-MGB |
| rs734186 | CCGTTGCTATCACCATCTGAAC | TGCACTGACTGTGAAATGCAAA | FAM-AGTTCAATTCAAACCAC-MGB | HEX-AGTTCAATCCAAACCAC-MGB |
| rs233716 | GCCTTCAGATAGACTGGGGATGT | CCCTCCCAATGGCTGAGTT | FAM-TTCTTCACGCCTTTC-MGB | HEX-CTTCTTCATGCCTTTC-MGB |
| rs4771856 | TGGTGCCCACTGCTCTGA | GAAATGAGTTAGGAGGCTACTGCAA | FAM-TTCACTCTACATTCAAC-MGB | HEX-TTCACTCTACCTTCAAC-MGB |
| rs12593223 | TGTGTGGCATTATTCCCTGGTA | CTTAAATGCCTGTGTTATTGAGCATG | FAM-CTCTGTCTACTTTATC-MGB | HEX-CTCTGTCTGCTTTATC-MGB |
| rs6499100 | TGAGGTTGTTGGTAGGAATACTGCTA | AGGTTGGGCCCTCTGTAATGT | FAM-AAGCCACGAGCAAT-MGB | HEX-AAAGCCATGAGCAAT-MGB |
