# Supplementary material for: Genetic Variants at 10p11 Confer Risk of Tetralogy of Fallot in Chinese of Nanjing
Source: PLoS One. 2014 Mar 3;9(3):e89636. doi: 10.1371/journal.pone.0089636 (PMC3940663; doi:10.1371/journal.pone.0089636)
Supplement: Table S4 — Associations of 7 SNPs with CHD in previous GWAS in Chinese population. (DOC) [file pone.0089636.s004.doc]

**Table S4:** Associations of 7 SNPs with CHD in previous GWAS in Chinese populaiton

| **Chr.** | **SNP** | **Subtypes** | **MAF ^b^** | | **OR (95% CI)^c^** | ***P* ^c^** |
| --- | --- | --- | --- | --- | --- | --- |
|  |  |  | **Cases** | **Controls** |  |  |
| 10p14 | rs1857231 | All cases | 0.17 | 0.19 | 0.88(0.76-1.04) | 0.13 |
|  | A/G ^a^ | ASD | 0.16 | 0.19 | 0.84(0.67-1.05) | 0.13 |
|  |  | VSD | 0.18 | 0.19 | 0.94(0.78-1.13) | 0.49 |
| 10p11.22 | rs2228638 | All cases | 0.09 | 0.09 | 1.00(0.81-1.23) | 0.99 |
|  | G/A ^a^ | ASD | 0.09 | 0.09 | 1.01(0.76-1.36) | 0.92 |
|  |  | VSD | 0.09 | 0.09 | 0.97(0.75-1.24) | 0.79 |
| 10p11.22 | rs734186 | All cases | 0.21 | 0.19 | 1.15(0.98-1.34) | 0.08 |
|  | T/C ^a^ | ASD | 0.21 | 0.19 | 1.14(0.92-1.41) | 0.25 |
|  |  | VSD | 0.21 | 0.19 | 1.11(0.93-1.34) | 0.25 |
| 12q24.13 | rs233716 | All cases | 0.37 | 0.35 | 1.06(0.94-1.21) | 0.34 |
|  | G/A ^a^ | ASD | 0.37 | 0.35 | 1.08(0.90-1.29) | 0.40 |
|  |  | VSD | 0.36 | 0.35 | 1.04(0.90-1.54) | 0.59 |
| 13q31.3 | rs4771856 | All cases | 0.32 | 0.32 | 1.02(0.89-1.16) | 0.80 |
|  | C/A^a^ | ASD | 0.31 | 0.32 | 0.98(0.81-1.17) | 0.80 |
|  |  | VSD | 0.33 | 0.32 | 1.03(0.89-1.20) | 0.68 |
| 15q13.3 | rs12593223 | All cases | 0.26 | 0.27 | 0.91(0.79-1.05) | 0.19 |
|  | G/A^a^ | ASD | 0.25 | 0.27 | 0.88(0.72-1.07) | 0.19 |
|  |  | VSD | 0.26 | 0.27 | 0.94(0.80-1.11) | 0.48 |
| 16q12.2 | rs6499100 | All cases | 0.21 | 0.21 | 0.99(0.86-1.15) | 0.94 |
|  | C/T ^a^ | ASD | 0.21 | 0.21 | 1.01(0.82-1.24) | 0.95 |
|  |  | VSD | 0.20 | 0.21 | 0.96(0.80-1.14) | 0.62 |

^a^ Major/minor alleles; ^b^ Minor allele frequency; ^c^ OR (95% CI) and *P* value derived from logistic regression analysis in additive model.
