# Supplementary material for: Genetic Variants at 10p11 Confer Risk of Tetralogy of Fallot in Chinese of Nanjing
Source: PLoS One. 2014 Mar 3;9(3):e89636. doi: 10.1371/journal.pone.0089636 (PMC3940663; doi:10.1371/journal.pone.0089636)
Supplement: Table S5 — Characteristics of CHD cases and non-CHD controls. (DOC) [file pone.0089636.s005.doc]

**Table S5:** Characteristics of CHD cases and non-CHD controls

| **Variables** | **Case (N=1,010)** | **Control (N=1,962)** | ***P*** |
| --- | --- | --- | --- |
| Age (mean±SD) | 4.59±11.05 | 4.63±11.34 | 0.38 |
| Sex |  |  | 0.94 |
| Male | 452 | 875 |
| Female | 558 | 1087 |
| Phenotype |  |  |  |
| ASD | 367 |  |  |
| VSD | 432 |  |  |
| TOF | 211 |  |  |
